# Bioprocessing of Functional Ingredients from Flaxseed

**DOI:** 10.3390/molecules23102444

**Published:** 2018-09-24

**Authors:** Christian Kwesi Ofotsu Dzuvor, Jordan Tauai Taylor, Caleb Acquah, Sharadwata Pan, Dominic Agyei

**Affiliations:** 1 Department of Chemical Engineering, Kwame Nkrumah University of Science and Technology, Kumasi ASH233, Ghana; chrisdzuv.cd@gmail.com; 2 Department of Food Science, University of Otago, Dunedin 9054, New Zealand; tayjo659@student.otago.ac.nz; 3 School of Nutrition Sciences, University of Ottawa, Ottawa, ON K1N 6N5, Canada; cacquah@uottawa.ca; 4 School of Life Sciences Weihenstephan, Technical University of Munich, Freising 85354, Germany; sharadwata.pan@tum.de

**Keywords:** bioprocessing, flaxseed, bio-refinery, bioactive compounds, functional food, nutraceuticals, detoxification

## Abstract

Flaxseeds (*Linum usitatissimum* L.) are oilseeds endowed with nutritional constituents such as lignans, lipids, proteins, fibre, carbohydrates, and micronutrients. Owing to their established high nutritional profile, flaxseeds have gained an established reputation as a dietary source of high value functional ingredients. Through the application of varied bioprocessing techniques, these essential constituents in flaxseeds can be made bioavailable for different applications such as nutraceuticals, cosmetics, and food industry. However, despite their food and health applications, flaxseeds contain high levels of phytotoxic compounds such as linatine, phytic acids, protease inhibitors, and cyanogenic glycosides. Epidemiological studies have shown that the consumption of these compounds can lead to poor bioavailability of essential nutrients and/or health complications. As such, these components must be removed or inactivated to physiologically undetectable limits to render flaxseeds safe for consumption. Herein, critical description of the types, characteristics, and bioprocessing of functional ingredients in flaxseed is presented.

## 1. Introduction

**Figure 1:**
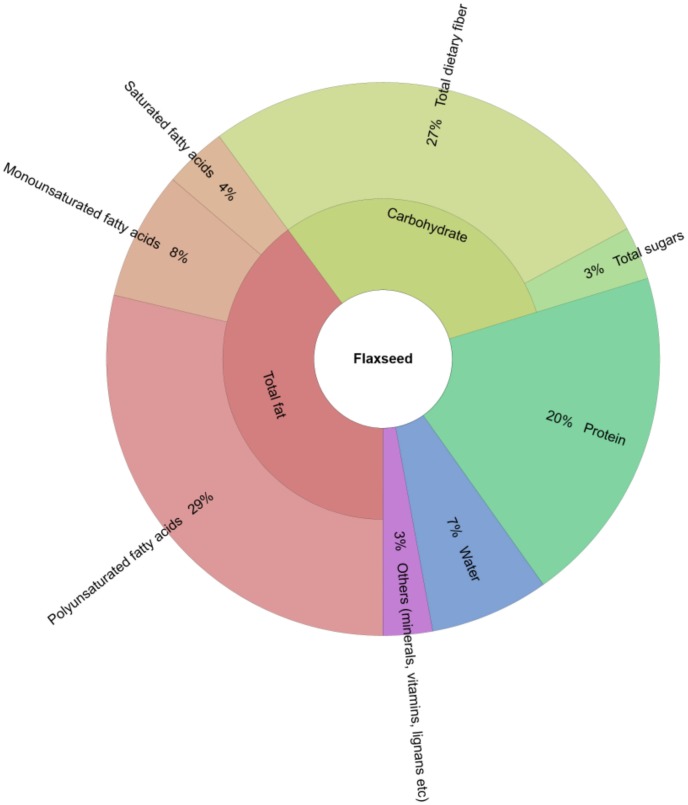
Krona chart showing the proximate composition of flaxseed. Source [22].

The common flax (or linseed, *Linum usitatissimum*), is an important agricultural commodity worldwide. It is considered a ‘superfood’ and generally recognized as safe (GRAS) source of vitamins, minerals, proteins and peptides (including bioactive cyclic peptides), lipids (including omega-3 and omega-6 polyunsaturated fatty acids), carbohydrates, lignans and dietary fibre [1,2,3,4,5,6], see Figure 1 for the composition of flaxseed). The health preventive and bioactive properties of these flaxseed components have been well studied. For example, the lipids, lignans, and fibre in flaxseed have been shown to have hypolipidemic, antiatherogenic, postprandial glycemic and insulinemic responses, anticholesterolemic and anti-inflammatory properties [7,8,9]. Moreover, other flaxseed components such as the proteins and peptides have been shown to induce certain desirable biologically active properties such as antioxidant, anti-inflammatory, antihypertensive, immune suppression/enhancement, glucose absorption control, etc. in living body systems [10,11,12,13,14]. It is therefore no wonder that flaxseed is included in many recipes in modern health foods culinary literature, especially after the publication of the outcome of the FlaxPAD clinical trials that studied “the effect of dietary flaxseed on improving symptoms of cardiovascular disease in patients with peripheral arterial disease” [15,16]. The global market of flaxseed is also growing; at a compound annual growth rate of 12%, the market is expected to be worth US$ 1.95 million by 2021 [17]. 

Not only does the diversity of biomolecules in flaxseed give this plant crop a high nutritional profile, but also, some of the flaxseed components have been explored as food additives due to certain functional properties they exhibit. Functional properties are those traits used to describe how the biochemical component of foods affects the sensorial properties of food during and after processing. For example, flaxseed mucilage has a high water-binding capacity [18], a property which is used to enhance the consistency, stability and viscosity of beverages [19]. Additionally, these mucilage have prebiotic potential (i.e., ability to modulate gut bacteria), and also provide bulking effect to stools thereby controlling constipation and irritable bowel syndrome, and body weight [20]. 

However, despite their food and health applications, flaxseed contains high levels of phytotoxic compounds, the consumption of which can lead to poorly bioavailable nutrients and/or health complications. The major antinutritive compounds in flaxseed are linatine, phytic acids, protease inhibitors and cyanogenic glycosides (linustatin, neolinustatin, linamarin and lotaustralin). Phytic acid interferes with the absorption of minerals such as calcium, zinc, magnesium, copper and iron [21], whereas, upon hydrolysis, cyanogenic glycosides release hydrogen cyanide, a respiratory inhibitor which in turn is converted to thiocyanates. Thiocyanates impede iodine uptake by thyroid gland and long term exposure exacerbates iodine-deficiency disorders such as goiter and cretinism [21]. Thus, all these antinutritive components must be removed or inactivated to physiologically undetectable limits to render flaxseed safe for consumption.

While flaxseed is one of the oldest oil crops used for food, information on the processing of its functional ingredients can vary. Moreover, many of the processing techniques developed for other oil crops are often not suitable for use on flaxseed. In this review, the functional/bioactive ingredients derived from flaxseed are described, while further elaborating on the processing techniques used for extraction or isolation of these ingredients. The potential challenges to the extraction of flaxseed-derived functional ingredients are raised, and ideas on potential avenues for more efficient, greener, extraction are also discussed. This review also highlights and discusses the strategies for the detoxification of flaxseed. 

## 2. Functional Ingredients in Flaxseed and Their Use in Food 

### 2.1. Lipids

Lipids are one of the high value functional ingredients in flaxseeds. Out of all the lipids available in flaxseed, the major component is the α-linoleic acid (~53%), followed by oleic acid (~19%), linoleic acid (~17%), palmitic acid (~5%) and stearic acid (~3%) [23,24]. The oil extracted from flaxseed contains a very high proportion of both mono- and polyunsaturated fatty acids (~91%), and minor amounts of saturated fatty acids (~9%). The high levels of unsaturated fatty acids is partly the reason why flaxseeds are considered to be a major health promoting agent [25]. In fact, for vegetarians, flaxseeds are the principal source of omega 3 fatty acids. Consequently, products containing flaxseed and its derivatives have been proposed as nourishing supplements for a range of dietary entities (see [26] and references therein). Several clinical studies have reported the extraordinary health benefits of alpha-linoleic acids [1,21]. This single constituent has been shown to control the development of atherosclerosis (mainly due to its constituents: eicosapentaenoic acid (EPA) and decosahexaenoic acid (DHA)), rheumatoid arthritis, inflammation, and asthma [10]. One significant advantage of α-linoleic acid has been its perennial positive influence on countering carcinogenic agents, thereby preventing development of malignant tumours and their metastases [27]. Some degree of protection against liver ailments is also provided by flaxseed lipids [21]. Additionally, significant lowering of low density lipoproteins (LDL, so called bad cholesterol) without noticeable changes in high density lipoproteins (HDL, or good cholesterol) has been linked to flaxseed consumption, in addition to nullification of lipid peroxidation [11]. 

### 2.2. Proteins, Peptides and Amino Acids

On average, flaxseed consists of ~21% proteins, and these proteins are mostly condensed in the cotyledons [4]. Flaxseed protein contains rich proportions of amino acids such as glutamic acid, methionine, arginine, cysteine and aspartic acid, with low amounts of lysine, threonine and tyrosine [4,21,28]. Several processing conditions do affect the final protein content of flaxseed products. Also, Flaxseed is not a complete source of dietary protein (due to its deficiency in certain essential amino acids like lysine), but nevertheless, the contributions that these beneficial proteins and bioactive compounds (mainly peptides) have, as well as their potential nutraceutical/nutritional applications have received huge attention in literature. [4,21,23]. 

The digestibility of flaxseed proteins is dependent on whether the protein is isolated in pure form or if it exists together with other nutritional components (mucilage, oils, etc.). Extracts of proteins are highly digestible (coefficient of digestibility value of 89.6%). Processing of flaxseed to remove oil and mucilage, thereby concentrating the proteins improved the in vitro digestibility of the proteins [29]. Moreover, flaxseed proteins also have a relatively high biological value (77.4%) [7].

Flaxseed proteins have been associated with antifungal properties [30], while distinct amino acids in flaxseeds, such as cysteine and methionine, have been shown to exhibit antioxidant characteristics [28]. Moreover, flaxseed protein hydrolysates have been reported to demonstrate anti-neurodegenerative properties by inhibiting nitric oxide synthesis [31], possesses anti-hypertensive properties by obstructing the transformation of angiotensin I to angiotensin II [12], demonstrates plasma glucose lowering abilities [32], amongst many others. 

**Figure 2:**
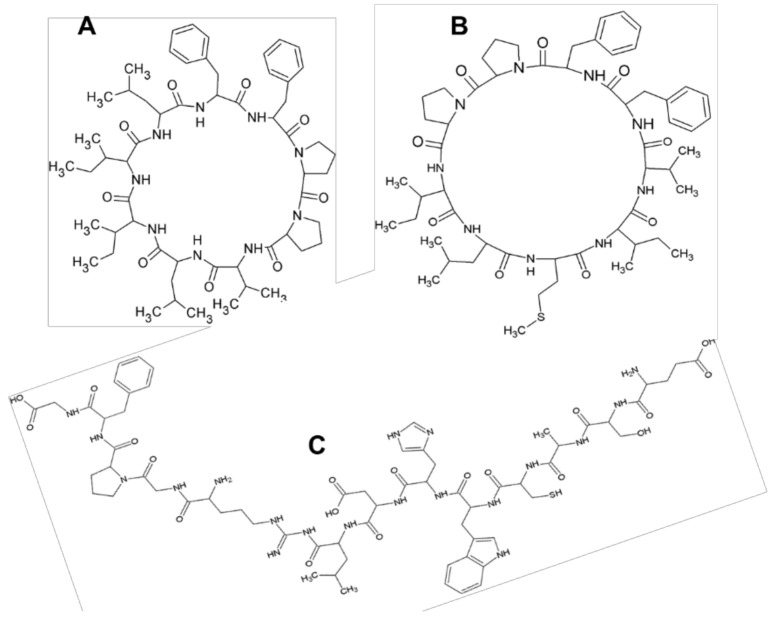
Chemical structures of some flaxseed peptides. (**A**) Cyclolinopeptide-A; (**B**) Cyclolinopeptide-B; (**C**) alcalase-derived antioxidative peptide.

Flaxseed is also one of the sources of cyclic peptides called cyclolinopeptides, and to date over 25 different kinds of these peptides have been identified in flaxseed. For example, the bioactive peptide cyclolinopeptide A (cyclo-(Pro-Pro-Phe-Phe-Leu-Ile-Ile-Leu-Val) and derivatives thereof has been shown to have antimalarial activity [33], immunosuppressive activity [3], as well as in activities in bone health (i.e., via osteoclast differentiation inhibition activity) [13]. Captured in Table 1 and Figure 2 respectively are some bioactive properties of some flaxseed hydrolysates and peptides, and chemical structures of some flaxseed peptides. 

### 2.3. Carbohydrates 

There is a lack of consensus in nutrition research on the levels of carbohydrate in flaxseed as well as whether this group of functional components significantly contribute to overall physiological nourishment through dietary consumption. Although flax as an entity is quite low in carbohydrates or sugars/starches (~1%) [23], the carbohydrate component of flaxseed has been reported to be ~29% [21]. This may be the reason why flaxseed is not considered to be an important source of food carbohydrates. Roughly, there are two major components of flaxseed polysaccharides: rhamnogalactouran and arabinoxylan. Rhamnogalactouran is an acidic polysaccharide, consists of L-fucose acid, L-rhamnose, D-galactouronic acid and d-galactose, and makes up about ~25% of flaxseed polysaccharides. Arabinoxylan, on the other hand is neutral, consists of arabinose, galactose and xylose, and makes up 75% of flaxseed polysaccharides [23]. It is worth noting that there is a significant difference of opinion regarding the productivity, exact constitution of monosaccharide components, and quality assessment of these polysaccharides [38]. Moreover, the use of carbohydrates in flaxseed as a functional food component need further investigations.

### 2.4. Dietary Fibers and Lignans and Other Components

**Figure 3:**
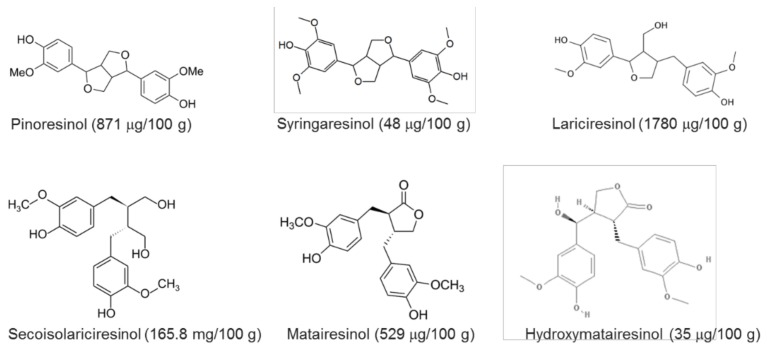
Chemical structures and amounts of some lignans in flaxseed. Lignin concentrations were taken from [42].

Dietary fibers and phenolic compounds (phenolic acids, flavonoids, and lignans) constitute a significant fraction of the flaxseed composition. A detailed discussion of the metabolism, composition and health properties of these components is available elsewhere [1,21] and references therein). Soluble flax mucilage finds frequent use as a food constituent, either as vegetable and fruit juice stabilizers, or as an ingredient for preventing syneresis and improving the texture of dairy products [4]. On the other hand, flaxseeds contain around 75 to 800 times larger portion of lignans compared to other cereal grains [21]. The most abundant lignin in flaxseed is secoisolariciresinol diglycoside [39]. Figure 3 shows the amounts and chemical structures of lignans in flaxseed. These phytoestrogens have been linked to several health promotion properties, including protection against cardiac and hepatic diseases, osteoporosis and carcinogens, and reduction of plasma cholesterol [21,40]. The in vitro and in vivo anti-inflammatory and antioxidant activity of flaxseed lignans has also been investigated [41]. 

### 2.5. Other Components (Vitamins and Minerals)

In addition to the major components, flaxseeds are also an important source of micronutrients such as vitamins and minerals, and phenolic compounds [21,23]. Amongst the vitamins, tocopherols (all the three forms: α, β, and γ) and niacins are found in abundant quantities in flaxseed. Around ~0.039% of vitamin E has been reported in flaxseeds [21], and their role in human health (e.g., antioxidant properties, protection against hypertension, cardiac ailments and Alzheimer’s disease) is also well documented [43]. Flaxseed contains high amounts of potassium (~5.6% to 9.2%), appreciable quantities of calcium (~0.25%), magnesium (~0.40%), phosphorus (~0.65%) and minute amounts of sodium (~0.027%) [44,45]. The high potassium means flaxseeds provide protection against stroke, helps promote free radical scavenging and inhibits platelet accumulation [45]. 

## 3. Bioprocess Techniques to Obtain Functional Flaxseed Ingredients

### 3.1. Processes for Extraction of Carbohydrates

Mucilage forms part of the waxy coat on the surface of flaxseed. Of interest are the hygroscopic carbohydrates that are a mixture of acidic and neutral polysaccharide fractions. The extraction and purification of this functional ingredient represent a time intensive process that is only commercially viable when coupled to the oil extraction process [46].

#### 3.1.1. Solid-Liquid Extraction

Solid-liquid extraction is the simplest technique for isolating bioactive carbohydrates and involves the mass transfer of solutes from a solid matrix into a solvent. Water is the primary solvent used in the extraction of carbohydrates [47]. Hot water extraction of mucilage involves the soaking of whole seeds or partially defatted seed cake. The resulting viscous crude extract is then precipitated using organic solvents or ultrafiltration with subsequent freeze drying. Whole seed extraction yields around 8% (*w/w*) and can be achieved by utilising 100 °C deionised water (1:10, g/mL) with stirring for 8 hours [48]. This yield can be further improved through milling of seed cake, however, the increased surface area exacerbates the uncontrolled release of proteins which is difficult to separate from the highly viscous mucilage and represents a serious issue for cost-effective commercial upscaling [49]. Activated charcoal and ion-exchange chromatography are the most utilised methods for the selective removal of carbohydrates from crude fractions, but these techniques are time consuming as one often needs several washing steps. Moreover, depending on the solvents used, removal of residues may also need to be undertaken. Solid-liquid extraction has the advantage of giving reasonable yields with little capital costs. The major disadvantage in using these methods is the laborious process of purification and slow mass transfer rate. Particularly when extracting carbohydrates from flaxseed, strategies such as increasing the surface area become a hindrance to extraction. Furthermore, the fact that organic solvents are routinely used to precipitate the mucilage, limits the use of this approach, especially when the aim is to reduce toxicity as the options of safe, cheap ‘green’ solvents are limited. 

#### 3.1.2. Pressurised Fluids

Based on the principles of Soxhlet, pressurised liquid (PL) extraction uses organic solvents for the rapid mass transfer of compounds from a solid matrix into the solvent [50]. Using high pressure and high temperature, the performance of solvents in solid-liquid extraction can be enhanced [51]. The rapid mass transfer of PL resolves the issues of conventional solid-liquid extraction. Ignoring the capital cost of implication, the disadvantages with using PL for the selective removal of bioactive carbohydrates are similar to that of conventional solid-liquid extraction.

#### 3.1.3. Sub/Supercritical Fluids

Little work has been done on the use of subcritical and supercritical fluid extraction of flaxseed polysaccharides. There is, however, an extensive body of work on the extraction of carbohydrates in bioprocessing. Carbohydrates are CO_2_-philic compounds [47]. While supercritical CO_2_ (scCO_2_) allows for the selective extraction of carbohydrates, organic modifiers need to be introduced which is not ideal when extracting bioactive carbohydrates [52,53]. However, the process is renewable and so using scCO_2_ is both a ‘greener’ and sustainable approach.

Subcritical fluid extraction is an adaption of PL, where the solvent utilised is water. With increased pressure and heat, water becomes less polar. The resultant process allows for the selective extraction of carbohydrates based on its solubility under different polarities of water [54]. This process doesn’t require the introduction of modifiers like scCO_2_ or organic solvents (as in the case of like traditional PL) and is therefore more suitable for food grade extracts of bioactive carbohydrates. Additionally, as subcritical fluid extraction has a greater range of polarities when compared to scCO_2_, this method has potential for greater selectivity [51,54,55].

#### 3.1.4. Ionic Liquids and Natural Deep Eutectic Solvents

Ionic liquids (IL) and natural deep eutectic solvents (NADES) represent an alternative to the use of water as a primary solvent in carbohydrate extraction and a replacement for traditional organic solvents [56]. Unlike other processes, the use of IL and NADES are a cost-effective alternative that is relatively non-toxic and easy to prepare [47,56]. IL are mixtures of organic salts that have low melting points and an immiscible vapour pressure. IL can be used to dissolve a range of carbohydrates from monosaccharides to polysaccharides. Adding small amounts of water to an IL allows for modification of the polarity and solubility of the solvent and target analyte. Choline chloride ILs produce the most effective preparation for the extraction of complex carbohydrates such as cellulose [47].

NADES are similar to IL, and choline chloride NADES are the most feasible options for carbohydrate extractions [56,57]. NADES are not only safer than IL but also cheaper. However, more investigation is needed to better identify and understand the toxicity of extracts prepared with NADES for human consumption. Moreover, the use of IL or NADES for the extraction of flaxseed components is almost non-existent. These represent the bottleneck to the commercial implementation of IL and NADES in this area [53].

### 3.2. Processes for Extraction of Proteins

Many fractionation techniques have been used over several decades for separation of seed protein of which flaxseed is no exception. Flaxseed proteins fall into three classes: albumin, globulin and glutelin [58]. Before their implementation as food component, protein must be extracted from seeds in order to enhance their digestibility, improve techno-functional properties as well as taste and colour, and reduce levels of antinutritive compounds [59]. Various protein extraction techniques have been proposed for the preparation of protein isolates from flaxseed. These include conventional approaches and novel processing technologies. The conventional isolation method includes alkaline/isoelectric precipitation, acid pre-treatment with ultrafiltration, and micellization technique (ammonium sulfate precipitation). Several novel technologies have also been discovered for protein extraction. These methods increases the yield, functionality and production sustainability of protein [60]. Novel technologies include ultrasound assisted extraction, electro-activation technique, pressurized low polarity water extraction, pulse–electric field technique, high voltage electrical discharge and complete or limited enzymatic (proteases) hydrolysis-assisted extraction. Before the application of the above-mentioned techniques, extraction of mucilage, gum removal, defatting and milling of flaxseed are employed to facilitate the protein recovery from the raw materials [61]. A description of some of these approaches is given below.

#### 3.2.1. Isoelectric Precipitation

Isoelectric precipitation is the most prevalently used approach for protein recovery and involves manipulating the pH of the protein solution to reach the solute pI that achieves precipitating of the proteins. With this extraction process, defatted flaxseed is suspended in water (usually at 1:10 or 20 *w/v* ratio), followed by alkaline extraction at high pH (pH 9–10 adjusted with base) and stirred for a few hour at a specified temperature (usually 37–40 °C) to extract proteins [62]. The extracted protein slurry is then centrifuged to recover both supernatant and residue. The residue is subjected to one more protein extraction step, followed by centrifugation and recovery as described above. The extracts are combined and adjusted to a low pH (usually 4.5–5) with acid at room temperature to precipitate the flaxseed proteins. The recovered protein precipitate is collected by centrifugation, and suspended in distilled water, after which the pH is adjusted to neutral. Furthermore, the protein solution can be lyophilized after dialysis to remove small molecules such as salt.

The use of isoelectric precipitation for protein extraction and recovery gives varying but relatively high yield (e.g., 48% by Gutiérrez, Rubilar, Jara, Verdugo, Sineiro and Shene [61]). However, this process has some drawbacks. These include creation of irreversible denaturation of protein by mineral acid [59], potential loss of functional properties (solubility and interfacial properties such as foaming and emulsification of protein), reduction of nutritional quality, and concentration of antinutritional components such as phytic acid [62].

#### 3.2.2. Partial Enzyme Hydrolysis-Assisted Extraction

With this approach, flaxseed meal is treated with proteases such as papain, trypsin, pancreatic, alcalase, pronase, ficin, unanizyme and flavouryme. Enzymatic hydrolysis of defatted and demucilaged flaxseed is initially adjusted to the pH optimal of the enzyme (e.g., alcalase (8.0), trypsin and pancreatin (7.5), flavouryme (7) and papain (6.5)) under mixing. The mixture is then incubated with one or a cocktail of enzymes where these proteases function to break the peptide linkage between adjacent amino acids and/or protein-carbohydrate molecules. The reaction mixture is stopped after several hours and protease is inactived by heating and rapid cooling. The suspension (mixture) is subjected to sonication, and the solubilized protein obtained by centrifugation and lyophilisation [63,64]. Partial enzymatic hydrolysis has several advantages such as helping to improve functional properties such as emulsification, foaming, water holding capacity and fat absorption capacity of the proteins [63]. In addition, this extraction method involves few side reaction, mild treatment condition, ease of control [65] and can help in the extraction of conjugated proteins such as glycoproteins. However, the use of enzymes can add to the overall cost of the processing method. Moreover, extensive hydrolysis of the proteins must be avoided. Thus, partial hydrolysis appear to be more beneficial in improving protein functionality than complete proteolysis, which can greatly impair protein functionality. 

#### 3.2.3. Micellization (Precipitation with Salts Such as Ammonium Sulfate) 

Micellization, as an isolation technique helps in the preservation of native state of protein and removal of non–protein components [60]. Protein precipitate by ‘salting out’ at different salt concentrations. The most commonly used salt is ammonium sulfate, due to its high solubility, minimal cost and lack of buffering capacity relative to other salts [66]. Micellization involves protein extraction with salt solution, usually in an ice bucket, followed by removal of insoluble material by centrifugation, recovery of precipitates by centrifugation, diafiltration to remove excess salts and final recovery by centrifugation [60,66]. Proteins extracted by micellization have low phytic acid and pentosans levels, higher enthalpies (meaning high structural order), and are lightly coloured [60]. However, ammonium sulphate precipitation can be onerous [66]. Micellization has been used to isolate flaxseed proteins to 93% yield [67].

#### 3.2.4. Acid Pre-Treatment with Ultrafiltration

This approach involves pre-treatment of flaxseed with acid followed by ultrafiltration [68]. Here, defatted flaxseed flour is suspended in water, adjusted and washed to pH 4.5 using mineral acids under mild agitation, and incubated for a time at room temperature. Acid extract and residue are separated through centrifugation of the protein slurry. The process is repeated several times on the residue to obtain more protein extracts which are later combined and filtered (ultrafiltration or diafiltration) to obtain retentate and permeate. The retentate is then spray–dried to obtain flaxseed protein. 

The advantages of ultrafiltration include improvement on the functional and nutritional characteristics of extracted protein [62]. It also leads to a product with low microbial counts. Use of acid pre-treatment/ ultrafiltration for the extraction of proteins from flaxseed has not been reported in the literature. However, Castel et al. [62] obtained protein yield of 10.2% for ultrafiltration of *Amaranth* sp. seeds.

### 3.3. Processes for Extraction of Lipids

Flaxseed is an oilseed which produces triglyceride oil that is rich in linolenic acid. Many techniques (conventional and novel) have been introduced for extracting oil from flaxseed. These include mechanical cold processing, solvent (polar and non-polar) extraction, supercritical fluid extraction, ultrasonic assisted extraction [69,70] and microwave assisted extraction [71].

#### 3.3.1. Cold Pressing

Cold press is a gentle mechanical oil extraction by which oil is forcibly pressed from seed through the application of pressure and shear force using screw press or oil expeller [72]. Prior to pressing, flaxseed is flaked, extruded and conditioned to remove all foreign material (dust, stone, immature seeds, etc.) to maximize operating capacity and oil recovery [70]. The cleaned and dried linseed is poured into an oil expeller or mechanical screw press with a rotating screw. During the operation of the screw press, the resolving screw forces the oil seed through a cylindrical enclosure, thus lessen the space occupied by the seeds, this leads to the expression of oil leaving behind the cake [73]. The extracted oil is then filtered to eliminate suspended solid using filtration equipment such as plate and flame filter. 

The main advantages of cold press extraction techniques are: low equipment cost and energy requirement, high quality and readymade consumable oil, and avoidance of usage of chemicals [71]. On the contrary, cold pressing has some drawbacks such as low yield, low content of vitamin, phospholipids, phytosterols, antioxidants and high oil content in the residual cake [70]. Seeping of oil-degrading enzymes such as lipoxygenase can degrade the extracted oil. Cold pressing is used to produce a flaxseed meal with a residual oil content ranging from 9% to 15% [70].

#### 3.3.2. Solvent Extraction

Different solvents are used for extraction of oil from oilseeds including flaxseeds. These solvents include acetone, methanol, petroleum ether, n-hexane dichloromethane, ethanol and heptane. Solvent extraction depends on factors such as nature of solvent, reaction time, temperature and solid/solvent ratio. With solvent extraction, cleaned, ground flaxseed is placed in a solvent in solid-to-solvent ratio of around 1:10 (*w/v*). The mixture is agitated and left to settle. Suspended solid is removed from micelle through vacuum filtration. Afterwards, the solvents are evaporated off to collect the extracted oil [69,74]. One of the highest recoveries of flaxseed oil reported in the literature was with hexane followed by dichloromethane [69]. However, hexane is known to be a hazardous solvent and this limits its use in food processing [71,75]. Heptane, iso-hexane, iso-propanol and ethanol have been recommended as the most promising substitute for oil extraction form oilseeds [69]. Solvent extraction has severe limitation including long extraction time, as well as disposal of large amounts of organic solvents which could be injurious to the health and the environment [69,71,76].

A novel technique that utilizes aqueous solvents (called aqueous extraction based on nitrogen protection) has also been trialled [77]. This process involves the use of salts solution to aid the separation of oils from aqueous mixtures by changing surface tension of the water phase as well as surface charges of components such as proteins and oils. This leads to a reduction in the number and stability of emulsions thereby enhancing oil extraction [78]. Salt-aided aqueous extraction is relatively low cost, and leads to maximum quantity and good quality of oil with enhanced stability against oxidation [79]. This method has been used to obtain high yield (86.4%) of transparent and clear oil from flaxseed [77].

#### 3.3.3. Microwave-and Ultrasound-Assisted Extraction 

Microwave and ultrasonic assisted extraction are two techniques used to improve oil extraction kinetics and efficiency [69,71]. These techniques improve oil extraction efficiency, by making it easier for solvents to penetrate into seed and increase contact surface between solvent and seed materials [69,80]. With these approaches, ground flaxseed is initially mixed with solvent followed by insertion of the ultrasonic probe into the mixture or placing the mixture in a microwave chamber. The oil sample is extracted under continuous ultrasonic/microwave energy and variant level of power output [69]. The advantages of microwave- and ultrasonic-assisted extraction include shorter extraction times, reduced solvent consumption and thermal damage to extracts, reduction in loss of bioactive compounds [81,82], as well as enhanced yield and economy [83]. On the contrary, it still involves the use of organic solvents which could be hazardous [71]. The ability of ultrasound and microwave to improve extraction of flaxseed oil has been studied and reported [81,84,85]. Long et al. [86] obtained about 68% oil recovery by coupling ultrasound treatment with immobilized enzyme cocktail consisting of cellulose, pectinase and hemicellulose. The oil obtained with this treatment had higher antioxidant properties and higher levels of unsaturated fatty acids when compared with oil obtained from organic solvent (hexane) extraction alone. Also, Ren et al. [87] obtained an extraction yield of 78.11% through pre-treatment of flaxseed with microwave energy, and the pre-treatment step had no effect on the fatty acid profile of oil extracted. 

#### 3.3.4. Sub/Supercritical Fluids Extraction

Sub/supercritical fluid (SFE) extraction processes have gained growing attention as industrial scale techniques in the recovery of edible oils from seeds such as canola, corn, sunflower, sorghum and flaxseed. This technique overcomes the shortcomings of solvent extraction since a gas at supercritical condition, rather than an organic solvent, is used in the extraction process. Some gasses used in supercritical fluid extraction include carbon dioxide, propane, toluene and ethane. Among these gases, carbon dioxide is the most commonly used due to its acceptable critical condition parameters, non-flammability, low cost, high selectivity and non-toxicity [76]. For the purpose of extracting oils from flaxseed by SFE techniques, ground flaxseed material is inserted in the extractor column to form a fixed bed of particle. The supercritical CO_2_ is then channeled through the bed and acts as a solvent that causes dissolution of oil and subsequent transition from the solid phase to the supercritical fluid phase [71,75,88]. 

**Figure 4:**
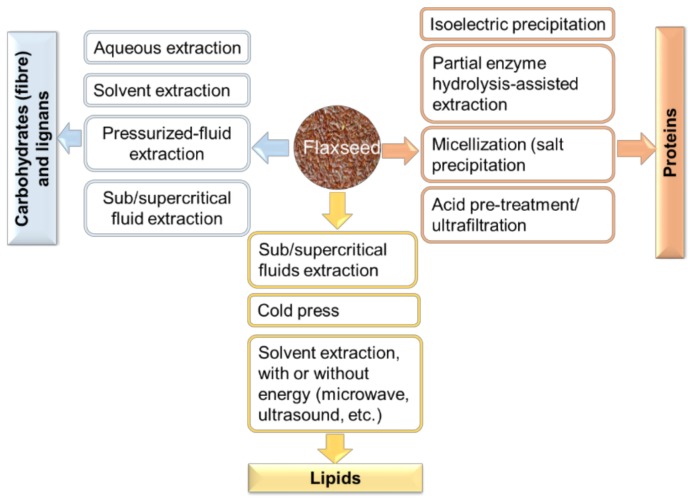
Processes for the biorefinery of flaxseed to obtained functional ingredients.

The main advantages of SFE are high quality of extract [76], green and eco-friendly approach [75], and industrial feasibility and scalability. However, its main drawback is in the high setup costs [75]. SFE has been used to extract oils from flaxseed and shown to give higher extraction efficiencies and high quality oil products rich in omega-3-fatty acid and omega-6-fatty acids as compared with other extraction techniques such as screw press and solvent extraction [89,90,91]. A simplified schematic of various techniques used to obtain functional ingredients from flaxseed is shown in Figure 4. 

## 4. Techniques for the Detoxification of Cyanogenic Glycosides

**Figure 5:**
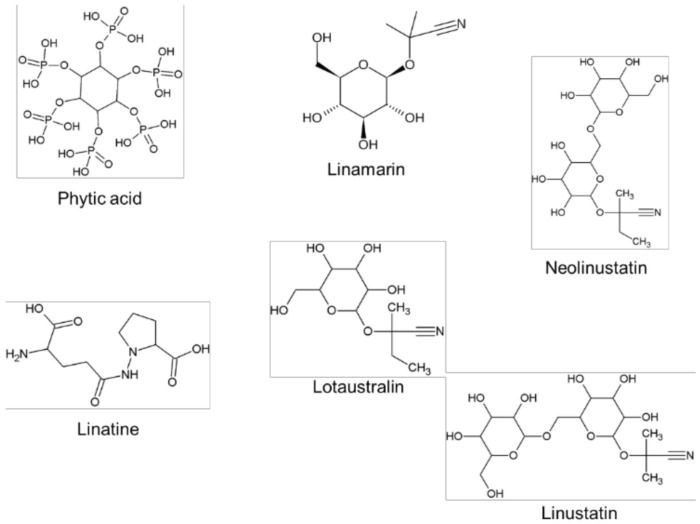
Chemical structures of flaxseed antinutritional compounds.

Processing of flaxseed has popularly been for their omega-3 fatty acids and lignans content, nevertheless, they also contain a rich amount of carbohydrate, minerals, and protein in abundance. However, the presence of antinutritional factors limits the bioavailability and bioaccessibility of the nutritional value derived therein. Flaxseeds contain antinutrients viz., linatine, protease inhibitors, phytic acids and cyanogenic glycosides (see Figure 5). These compounds affect the bioavailability and bioaccessibility of essential nutrients, as well as triggers life-threatening effects. For instance, the impact of linatine as an antinutritional factor in flaxseeds, causing symptoms of vitamins B_6_ deficiency, was observed in fed chicks as early as 1946 [92]. Nevertheless, the observed negative impact is yet to be observed in humans. Phytic acids act as antinutritional factors in flaxseeds by chelating minerals such as copper, zinc, magnesium, iron, selenium and calcium present, thereby inhibiting their release. Cyanogenic glycosides are nitrogen-containing secondary metabolites which when ingested can be hydrolysed to produce hydrogen cyanide, a highly toxic chemical [93]. Chronic exposure to hydrogen cyanide toxins leads to defects in the neurological, respiratory, and cardiovascular system as well as the thyroid gland [94]. There are four types of cyanogenic glycosides found in flaxseeds, namely; linustatin, neolinustatin, lotaustralin and linamarin [5,95]. Owing to their toxicity, cyanogenic glycosides are critical antinutritional factors that need to be removed from flaxseeds to enhance their health benefits. 

### 4.1. Solvent Extraction

Solvent extraction method is one of the traditional means of removing cyanogens in flaxseeds. A two-phase solvent extraction system comprising alkanol-ammonia-water/hexane has been demonstrated in the extraction of oil and detoxification of flaxseed meals [96]. An optimum composition of an alkanol-ammonia-water system for detoxification of flaxseeds was obtained by using 95% methanol, 10% ammonia, 10–15% water [97]. The aforementioned composition resulted in a cyanogen detoxification yield of about 56% in the first run of extraction, 80% in the second run and over 90% in the third run. Similarly, Varga et al. [98] used a two-phase solvent system consisting of 0.08% NaOH, 10% water, 90% methanol to extract about 83.6% cyanogens present in flaxseeds. However, the challenge with solvent extraction is the inability to completely detoxify flaxseeds of the cyanogens, and concomitantly reducing the protein, fibre, fat, and lignin content [99].

### 4.2. Heat Treatment

Thermal treatment via oven, steam or sun heating of foods containing cyanogenic glucosides have previously been shown to be effective in detoxifying the food to acceptable detection limits [99,100,101]. The disadvantages of using steam heat in detoxification of flaxseeds are the loss of nutritional constituents such as lignans and fatty acids in flaxseeds, high energy consumption and carbon footprint [102]. High thermal treatment, above 120 °C, have also been shown to be less efficient resulting in the retention of a substantial amount of the cyanogens in the flaxseed [99]. This is as a result of the inactivation of endogenous β-glucosidases which have the ability to degrade indigenous cyanogens. An alternative approach proposed by Yamashita et al. [99] involves pulverising flaxseeds into a powder which can then be incubated at about 30 °C and an optimised time to completely degrade the cyanogens to release hydrogen cyanides. Hydrogen cyanides released into the slurry were then steam evaporated to yield a cyanide-free dry flaxseed meal without adversely affecting the protein, fibre, fat, and lignin.

### 4.3. Biological Treatment 

The use of a biological approach in the degradation of hydrogen cyanide is a relatively cheaper approach in food processing. Commercial enzymes such as linamarase, xylanase, and cellulase have been successfully demonstrated in the degradation of cyanogenic glucosides in cassava and cassava-based food products to acceptable limits [103,104]. Contrary, Yamashita et al. [99] observed that flaxseeds had a low substrate specificity after experimenting on similar commercial enzymes linamarase from *Phaseolus lunatus* and β-glucosidase from *Prunus dulcis* which yielded low activity. Wu et al. [102] utilised a response surface methodology to optimise a fermentation process to detoxify flaxseeds of cyanogenic glucosides. The optimised fermentation process involved the use of 12.5% β-glucosidase and 8.9% cyanide hydratase enzymes to reduce cyanide concentration in flaxseed powder from 1.156 to 0.015 mg after 48 h. 

## 5. Conclusion and Future Outlook 

Flaxseed is a nutrient-and functional food ingredient-rich crop owing to the high levels of vitamins, minerals, proteins, lipids, dietary fibre and lignans. The food and nutritional applications of flaxseed will continue to attract the attention of health-conscious consumers and food manufacturers alike. In this regard, obtaining high value ingredients/component through bio-refinery of flaxseed (where various nutrients and functional ingredients are extracted from seeds along the process chain) or through valorisation of by-products (example extraction of proteins and fibre/mucilage from flaxseed cake derived from oil extraction processes) is very attractive. However, to explore the full potential of this ‘super food’, further research is needed specifically in the design of industrially and economically feasible, food-grade, ‘green’ approaches for the extraction and purification of the many functional food ingredients obtainable from flaxseed. The suitability of extraction procedure and isolation techniques depend on the type of the nutrient, the scale of isolation, and commercialization feasibility, amongst others. It must be noted that no technique is perfect and each method has its own intrinsic benefits and disadvantages. For isolation of carbohydrates from flaxseeds, solid-liquid extraction may be adopted for reasonably high yields and low costs. However, super/subcritical fluid extraction strategies have garnered considerable attention of late due to their assorted range of benefits such as renewability and sustainability, varied range of polarities, higher selectivity, which are all suitable for derivation of food-quality extracts. For extraction of proteins from flaxseeds, both conventional and novel strategies have been tried. In spite of certain demerits, isoelectric precipitation is still the most prevalent traditional method for protein isolation, mainly because of the associated high yield. Novel techniques such as ultrasound-assisted extraction, electro-activation and pressurized low-polarity water extraction also hold promise, but these have to be optimized to improve their performance and overcome the associated high maintenance costs. For lipid extraction, aqueous extraction with conferred nitrogen protection has emerged to be a technique of interest, considering its extremely high production yield. Although linked with high costs, sub/supercritical fluid extraction of lipids remains a high value technique because of the high quality of the extract, amenability to environment, scalability and commercial feasibility. 

In order to get the correct nutritional balance in food, there has been a growing trend where the consumption of the whole food (i.e., intact seeds) is encouraged, as opposed to the intake of extracted food components (in the form of nutraceuticals). This approach is laudable, but its practice is limited by the fact that some foods contain indigenous antinutritives that must be removed or deactivated before the food can be consumed. Thus, appropriate means of removing or reducing the levels of the antinutritives (especially the cyanogenic glycosides) in intact flaxseed needs to be given priority. Altogether, flaxseed is an excellent plant food candidate that meets the needs of the 21st century consumer in terms of being rich in nutrients as well as bioactive and functional ingredients. 

## Figures and Tables

**Table 1:** Primary structures, method of production, and bioactive properties of some flaxseed hydrolysates and peptides.

Type	Structure or Primary Sequence	Production Method	Biological Property	Reference(s)
**Peptide**	Gly-Phe-Pro-Gly-Arg-Leu-Asp-His-Trp-Cys-Ala-Ser-Glu	Hydrolysis by Alcalase	Antioxidant	[34]
**Hydrolysate**	<1 kDa peptides fractions	Hydrolysis by protease from *Bacillus altitudinis* HK02	Antimicrobial activity	[35]
**Hydrolysate**	1–3 kDa peptides fractions	Hydrolysis by protease from *Bacillus altitudinis* HK02	Antioxidant activity	[35]
**Hydrolysate**	Less than 4 kDa peptide fractions	Hydrolysis by thermolysin and pronase	Antioxidant; Antihypertensive (angiotensin I-converting enzyme, ACE-inhibitory) activity	[36]
**Hydrolysate**	Less than 1, and 1–3 kDa peptide fractions	Hydrolysis by thermoase and membrane ultrafiltration	Antihypertensive (ACE-inhibitory); Renin-inhibitory activity	[37]
**Cyclolinopeptide -A**	cyclo-(Pro-Pro-Phe-Phe-Leu-Ile-Ile-Leu-Val)	Extraction	Immunosuppressive activity; Antioxidant; Antimalarial activity	[13,14]
**Cyclolinopeptide -B**	cyclo-(Pro-Pro-Phe-Phe-Val-Ile-Met-Leu-Ile)	Extraction	Immunosuppressive activity	[13]
**Cyclolinopeptide -E**	cyclo-(Pro-Leu-Phe-Ile-MetO-Leu-Val-Phe)	Extraction	Immunosuppressive activity	[14]

## Supplementary Materials

An interactive and comprehensive version of Figure 1 is available at this link: https://dl.dropboxusercontent.com/s/5o729rqawjuci5s/Krona%20for%20composition.html?dl=0. Readers are encouraged to download this html file onto a local drive before opening.
